# Refinement of Replacement Fluid Using Human Serum Albumin Preparation to Enhance Biocompatibility in Therapeutic Plasma Exchange

**DOI:** 10.1002/jca.70077

**Published:** 2025-11-26

**Authors:** Yuki Narita, Yoshitaka Takegawa, Tomoyuki Mizuta, Katsuji Otsuka, Hirotoshi Nakano, Daisuke Kadowaki, Hideyuki Saito, Masataka Adachi

**Affiliations:** ^1^ Department of Clinical Pharmaceutical Sciences, Graduate School of Pharmaceutical Sciences Kumamoto University Kumamoto Japan; ^2^ Department of Pharmacy Kumamoto University Hospital Kumamoto Japan; ^3^ Faculty of Pharmaceutical Sciences Sojo University Kumamoto Japan; ^4^ Immune inflammation & Hematology Group, Medical Affairs Section, R&D Administration Division KM Biologics Co. Ltd. Kumamoto Japan; ^5^ Department of Medical Engineering Center Kumamoto University Hospital Kumamoto Japan; ^6^ Department of Nephrology Kumamoto University Graduate School of Medical Kumamoto Japan; ^7^ Kidney Disease and Dialysis Center Kumamoto University Hospital Kumamoto Japan

**Keywords:** albumin, calcium concentration, replacement fluid, therapeutic plasma exchange

## Abstract

Human serum albumin (HSA) solution is widely used as a replacement fluid during therapeutic plasma exchange (TPE). However, the ionic concentrations in commercially available 5% HSA solutions differ significantly from physiological levels. In this study, we developed a simple method for preparing a replacement fluid with ion concentrations closer to physiological norms by mixing 25% HSA with lactated Ringer's solution, sodium chloride, and calcium gluconate hydrate. We prepared three types of replacement fluids, each with a 4.0% albumin concentration and total volumes of 1.83, 2.43, and 3.04 L, and confirmed their compositional stability for up to 48 h. Compared with the standard 5% HSA solution, the prepared fluids had calcium, sodium, potassium, and chloride levels more closely aligned with physiological values. Using HSA‐based replacement fluids with preadjusted electrolyte concentrations may help reduce the risk of imbalances, such as hypocalcemia, during TPE.

## 1 Introduction

Therapeutic plasma exchange (TPE) is a procedure in which plasma is separated from blood cells and removed, then replaced with a substitute fluid to eliminate pathogenic substances. Its effectiveness has been demonstrated in a wide range of diseases [1]. The choice of replacement fluid among human serum albumin (HSA) solution, electrolyte solutions, and fresh frozen plasma (FFP) is based on the underlying disease and a careful evaluation of the risks and benefits of each option. In general, albumin is the first choice for TPE because most indications do not require supplementation of coagulation factors, and it offers fewer side effects, wide availability, comparable therapeutic effects to FFP, reduced risk of adverse reactions, and lower infection risk [2]. The commercially available isotonic 5% solution is a commonly used albumin replacement fluid [3], but one significant issue is the fact that its electrolyte concentrations differ from those of normal plasma. In particular, given that hypocalcemia is one of the most common adverse effects of TPE [4], the absence of calcium in 5% isotonic albumin solutions cannot be overlooked. Moreover, excessive administration of sodium and chloride has been reported [5]. To address these issues, a method of preparing replacement fluid by mixing 25% hypertonic albumin with Ringer's lactate (RL) and 10% sodium chloride solutions has been reported [6]. However, this method requires trained personnel and adequate preparations for individual adjustments of the electrolyte concentrations for each patient. Although diluting hypertonic albumin is expected to yield replacement fluids that match the osmolality and electrolyte concentrations of the patient's serum, customizing the concentrations for each patient may be difficult to implement in all hospitals. We believe that a balance between physiological compatibility and practical simplicity in clinical settings is essential.

Our objective was to establish a new method for preparing replacement fluid that addresses these challenges. In this study, we formulated replacement fluids, which had minimal mismatch with patient plasma, using 25% hypertonic albumin and a simple preparation procedure. The prepared replacement solutions were measured and evaluated in terms of the various components, including their changes over time.

## 2 Materials and Methods

### 2.1 Composition of the Replacement Fluid

This study was designed as a preliminary evaluation of a clinical preparation method rather than a comprehensive manufacturing validation compliant with good manufacturing practice (GMP) standards. The focus was on assessing the feasibility and short‐term stability of a simplified preparation method suitable for hospital pharmacy settings. To simplify the method and reduce the risk of errors during preparation of replacement fluids in the clinical setting, each formulation was intended to be used in its standard full‐volume unit. The combination of the number of units was designed to approximate the normal serum range in healthy individuals. In addition, whenever possible, high‐volume standard formulations were selected to minimize the effort required for preparation. First, the largest available formulation of 25% HSA solution (Kenketsu Albumin 25% IV Injection 25 g/100 mL “KMB,” KM Biologics Co. Ltd., Kumamoto, Japan) was selected as the replacement fluid base. For dilution, 500 mL of RL solution (Lactec Injection; Otsuka Pharmaceutical, Tokushima, Japan) was selected based on the dilution ratio of albumin. To adjust the osmotic pressure and electrolyte concentrations, 20 mL of 10% sodium chloride injection (Nissin; Nissin Pharmaceutical Co. Ltd., Yamagata, Japan) and 10 mL of 8.5% calcium gluconate hydrate injection (Calcicol; Nichi‐Iko Pharmaceutical Co. Ltd., Toyama, Japan) were added. The concentrations of the components of each preparation are listed in Table 1. The number of units of each preparation used according to the prepared replacement fluid volume is shown in Table 2. The basic compositions had volumes of 1830 mL for No. 1; 2430 mL for No. 2; and 3040 mL for No. 3. For volumes larger than these, combinations of the three compositions were used. The theoretical concentrations of each component in Nos. 1–3 are shown in Table 3. The composition was determined based on high biocompatibility with the normal serum range among healthy Japanese individuals [7].

**TABLE 1:** List of the various components of the albumin replacement fluid.

	Albumin concentration	Calcium concentration	Sodium concentration	Potassium concentration	Chloride concentration
Kenketsu albumin 25% IV injection 25 g/100 mL “KMB”	12.5 g/50 mL	—	2.0 mg/mL (approx. 87 mEq/L)	—	0.2 mg/mL
Ringer's lactate solution (lactec injection)	—	3 mEq/L	130 mEq/L	4 mEq/L	109 mEq/L
10% sodium chloride injection	—	—	34 mEq/20 mL	—	34 mEq/20 mL
Calcicol injection 8.5%	—	7.85 mg/mL (0.39 mEq/mL)	—	—	—

*Note:* Values were cited from the package inserts of each product.

**TABLE 2:** Preparation of the albumin replacement fluids.

Composition	Total volume (mL)	Kenketsu albumin 25% IV injection 25 g “KMB” (100 mL)	Ringer's lactate solution (500 mL)	10% sodium chloride injection (20 mL)	Calcicol injection 8.5% (10 mL)
No. 1	1830	3 vials	3 bottles	1 ampule	1 ampule
No. 2	2430	4 vials	4 bottles	1 ampule	1 ampule
No. 3	3040	5 vials	5 bottles	1 ampule	2 ampules
No. 1 + No. 1	3660	6 vials	6 bottles	2 ampules	2 ampules
No. 1 + No. 2	4260	7 vials	7 bottles	2 ampules	2 ampules
No. 2 + No. 2	4860	8 vials	8 bottles	2 ampules	2 ampules
No. 2 + No. 3	5470	9 vials	9 bottles	2 ampules	3 ampules

*Note:* Based on the 100 mL standard of 25% albumin, combinations were devised on the premise of using up each preparation.

**TABLE 3:** Theoretical concentrations of each component of the albumin replacement fluid.

Unit	Albumin	Calcium	Sodium	Potassium	Chloride
	g/dL	mg/dL	mmol/L	mEq/L	mEq/L
Composition No. 1 (1830 mL)	4.1	9.2	139	3.3	109
Composition No. 2 (2430 mL)	4.1	8.2	135	3.3	105
Composition No. 3 (3040 mL)	4.1	10.1	133	3.3	102
Reference range^a^	4.1–5.1	8.8–10.1	138–145	3.6–4.8	101–108

^a^ Based on the Japanese Society of Laboratory Medicine (2021). Guidelines for Clinical Laboratory Testing JSLM2021. Tokyo: Japanese Society of Laboratory Medicine.

### 2.2 Procedure

**FIGURE 1:**
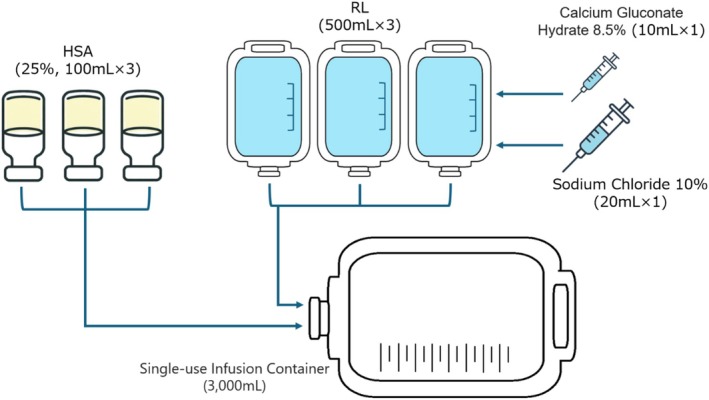
Preparation of albumin replacement fluid composition no. 1 (1830 mL).

After transferring 10% sodium chloride and 8.5% calcium gluconate solutions into one RL container, all contents of the RL and HSA solutions were transferred into a 3000‐mL single‐use infusion container (Alimebag α; Nipro Corporation, Osaka, Japan) (Figure 1).

### 2.3 Measurement Method

Three packs each of the three replacement fluid compositions (Nos. 1, 2, and 3) were prepared. To assess temporal changes, three measurements were taken from each of the three packs as part of measurement error risk management (*N* = 3) at four time points (i.e., 1, 6, 24, and 48 h) after preparation. The median of the three measurements from each pack was then used to calculate the mean ± standard deviation of these three median values. As a control group, three vials of 5% HSA solution (Albumin 5% IV 12.5 g/250 mL “JB;” Japan Blood Products Organization, Tokyo, Japan) were used and measured in the same manner. However, because this commercial product does not require mixing, time‐dependent changes were not assessed. The items measured were concentrations of electrolytes (sodium, potassium, chloride, and calcium); albumin concentration; pH; osmolality; and turbidity. Sodium, potassium, and chloride were measured using an electrolyte analyzer (EA07; A&T Corporation, Kanagawa, Japan) and the ion‐selective electrode method. Albumin and total calcium were measured using an automated clinical analyzer (7180 Clinical Analyzer; Hitachi High‐Tech Corporation, Tokyo, Japan) and the BCG and MXB methods, respectively. pH was measured using a pH meter (F‐21; HORIBA, Kyoto, Japan) and the glass electrode method. Osmolality was measured using an osmometer (OSMOMAT 030‐D‐RS; GONOTEC GmbH, Berlin, Germany) and the freezing point depression method. Turbidity was measured using a microplate reader (Infinite M200; Tecan Group Ltd., Männedorf, Switzerland) at an absorbance of 450 nm, which was selected based on preliminary testing to minimize the influence of absorbance peaks of the replacement fluid components. The ionized calcium concentration was measured only at the 1‐h time point using a blood gas analyzer (RAPIDLab 1265; Siemens AG, Munich, Germany).

## 3 Results

All three types of albumin replacement fluid had no significant deviations from the theoretical values and were confirmed to have compositions with minimal mismatch with patient sera (Table 4). In compositions No. 1, 2, and 3, slight variations in the measured values were observed over time in some measurements (Tables 4 and 5). As previously reported, the control 5% HSA solution was confirmed not to contain calcium and to have high sodium and chloride contents (Table 4).

**TABLE 4:** Concentrations of albumin and various ions in the replacement fluids (*N* = 3).

		Albumin	Total calcium	Ionized calcium	Sodium	Potassium	Chloride
		g/dL	mg/dL	mmol/L	mmol/L	mEq/L	mEq/L
Composition No. 1 (1830 mL)	1 h	4.0 ± 0.00	8.5 ± 0.21	1.3 ± 0.02	140 ± 0.6	3.4 ± 0.00	108 ± 0.6
	6 h	4.0 ± 0.00	8.5 ± 0.10	—	140 ± 0.6	3.4 ± 0.00	107 ± 0.6
	24 h	4.0 ± 0.05	8.3 ± 0.15	—	139 ± 0.6	3.4 ± 0.00	107 ± 0.0
	48 h	4.0 ± 0.00	8.3 ± 0.15	—	139 ± 0.0	3.3 ± 0.06	107 ± 0.6
Composition No. 2 (2430 mL)	1 h	4.0 ± 0.06	7.8 ± 0.06	1.2 ± 0.01	136 ± 0.0	3.4 ± 0.00	103 ± 0.6
	6 h	4.0 ± 0.06	7.8 ± 0.06	—	136 ± 0.6	3.4 ± 0.00	103 ± 0.6
	24 h	4.0 ± 0.00	7.7 ± 0.06	—	136 ± 0.6	3.4 ± 0.00	103 ± 0.6
	48 h	4.0 ± 0.00	7.5 ± 0.12	—	134 ± 0.0	3.3 ± 0.00	103 ± 0.6
Composition No. 3 (3040 mL)	1 h	4.0 ± 0.00	9.6 ± 0.00	1.4 ± 0.00	133 ± 0.0	3.4 ± 0.00	101 ± 0.6
	6 h	4.0 ± 0.06	9.6 ± 0.06	—	133 ± 0.0	3.4 ± 0.00	100 ± 0.6
	24 h	4.0 ± 0.00	9.5 ± 0.06	—	133 ± 0.0	3.4 ± 0.00	101 ± 0.6
	48 h	4.0 ± 0.00	9.3 ± 0.06	—	131 ± 0.6	3.3 ± 0.00	100 ± 0.0
5% HSA	—	5.2 ± 0.06	0.0	0.0	149 ± 0.0	0.2 ± 0.00	133 ± 0.0

*Note:* The mean ± standard deviation of the median values from three packs is listed.

**TABLE 5:** pH, osmotic pressure, and turbidity of the replacement fluids (*N* = 3).

		pH	Osmotic pressure (mOsm/kg)	Turbidity
Composition No. 1 (1830 mL)	1 h	6.94 ± 0.010	273 ± 2.9	0.060 ± 0.0015
	6 h	6.98 ± 0.023	275 ± 2.5	0.060 ± 0.0002
	24 h	7.00 ± 0.038	273 ± 0.6	0.060 ± 0.0006
	48 h	6.96 ± 0.023	276 ± 2.1	0.061 ± 0.0017
Composition No. 2 (2430 mL)	1 h	6.94 ± 0.006	264 ± 1.2	0.060 ± 0.0005
	6 h	6.96 ± 0.006	265 ± 1.7	0.060 ± 0.0003
	24 h	6.98 ± 0.017	267 ± 2.1	0.059 ± 0.0005
	48 h	6.95 ± 0.012	268 ± 1.0	0.061 ± 0.0031
Composition No. 3 (3040 mL)	1 h	6.95 ± 0.000	262 ± 0.6	0.060 ± 0.0001
	6 h	6.96 ± 0.006	261 ± 0.6	0.060 ± 0.0007
	24 h	6.98 ± 0.010	261 ± 1.0	0.059 ± 0.0006
	48 h	6.95 ± 0.012	264 ± 2.1	0.064 ± 0.0095
5% HSA	—	6.80 ± 0.026	280 ± 1.0	0.090 ± 0.0003

*Note:* The mean ± standard deviation of the median values from three packs is listed.

## 4 Discussion

In this study, we demonstrated that a highly biocompatible albumin replacement fluid can be prepared using a simple procedure. Although the concentrations of some electrolytes were slightly outside the reference values in normal subjects, the prepared albumin replacement fluids were closer to normal than the widely used 5% albumin solution. We believe that these compositions could potentially contribute to improved electrolyte management during TPE.

Hypocalcemia is a common adverse effect during TPE and is often due to the use of FFP, which contains citrate that chelates calcium in the bloodstream. Moreover, citrate is typically used as the anticoagulant during centrifugation‐based TPE [8]; therefore, even when a commercially available 5% albumin solution is used instead of FFP, the risk of calcium chelation remains. Warner et al. reported that hypocalcemia occurred in 44.1% of 145 patients when 5% albumin was used as the replacement fluid in the majority of 1219 centrifugation‐based TPE procedures with citrate anticoagulation [9]. Conversely, Kankirawatana et al. reported that continuous calcium gluconate infusion reduced the incidence of hypocalcemia to 2.7% [10]. The TPE: Core Curriculum 2023 published in the *American Journal of Kidney Diseases* also recommended oral or intravenous calcium supplementation when citrate is used [8]. During membrane filtration‐based TPE, the anticoagulant chosen is generally heparin [8], which is believed to decrease the risk of hypocalcemia, compared with citrate. However, because commercial 5% albumin solutions do not contain calcium, a decrease in both ionized and total calcium concentrations is unavoidable [11]. Albumin has a strong binding affinity to ionized calcium in plasma, and calcium is removed when manufacturing albumin products [10]. Therefore, in addition to the dilutional effect, albumin binding to calcium in the patient's blood may further lower ionized calcium levels and pose an additional risk for hypocalcemia [12]. Although reports evaluating the risk of hypocalcemia when using 5% albumin without citrate (i.e., with heparin anticoagulation) as a replacement fluid are limited, a case series reported asymptomatic hypocalcemia during TPE in one of seven pediatric patients [13].

Hypokalemia during TPE has also been reported, with an incidence as high as 36.6% [9, 10, 14, 15]. Kankirawatana et al. reported a significant decrease of 7% in average potassium levels after TPE, despite the routine addition of potassium chloride to the 5% albumin solution [10]. Although citrate‐induced metabolic alkalosis has been suggested to cause hypokalemia [16], the study by Kankirawatana observed a decrease in potassium levels even in the absence of alkalosis, indicating that the dilutional effects of the replacement fluid should also be considered. In any case, because TPE involves the infusion of large volumes of replacement fluid, electrolyte balance must be carefully managed. Current TPE strategies, particularly for calcium, include real‐time monitoring and calcium supplementation as needed or by continuous infusion, depending on the facility. Preadjusting the electrolyte composition of the replacement fluid itself to match physiological levels may further enhance the safety of TPE and theoretically help optimize electrolyte management during TPE, in terms of homeostasis not only of calcium but also of other electrolytes.

The replacement fluid used in this study contained albumin at a concentration of 4.0 g/dL, which is lower than the typical 5.2 g/dL found in standard 5% albumin solutions and may offer potential clinical advantages. A 4.0 g/dL albumin solution has an osmolality closer to that of normal human plasma, making it less likely to cause abrupt shifts in intravascular volume and thereby helping to maintain hemodynamic stability. Using a replacement fluid that more closely matches physiological conditions may be particularly beneficial for patients with hypoalbuminemia (albumin < 4.0 g/dL) prior to TPE.

In this study, preparation of the replacement fluids was simplified by designing the combinations based on the complete use of existing commercial formulations. For example, in the early days of total parenteral nutrition (TPN) therapy, customized mixing was performed for each patient. However, due to the complexity of preparation and safety concerns, such as compounding errors, commercially available preformulated TPN solutions were eventually developed in Japan after standardizing the composition of the solutions [17]. Similarly, the standard compositions evaluated in this study may serve as a reference and potentially contribute to the future development of dedicated replacement fluids for TPE.

One important consideration when preparing this replacement fluid was the variation in osmolarity and electrolyte concentrations among different manufacturers of the albumin product [5]. Therefore, the dilution method must be adjusted according to the specific formulation of each manufacturer. In this study, we prepared the replacement fluid by diluting 25% albumin from KM Biologics. If albumin products from a different manufacturer will be used in the future, the theoretical calculations and actual measurements must be reevaluated. Although the preparation of albumin replacement fluid is simple, precautionary measures to avoid errors must be taken when manually transferring the required number of vials into a single‐use infusion container according to the protocol. We created a checklist for the preparation and attached it as a label on the single‐use infusion container. This approach may help prevent simple mistakes and allows the requesting medical staff to perform a double‐check. In clinical practice, additional safety can be ensured by weighing the prepared replacement fluid, thereby establishing a more reliable verification system.

Regarding the stability of the replacement solution, particular attention was paid to the behavior of calcium. Although calcium‐containing solutions are known to carry a risk of precipitation [18] and their affinity for albumin is well documented [19]—raising concerns about potential effects—and a slight change in total calcium concentration was observed between the 1‐ and 48‐h measurements, no visible precipitation was detected in this formulation. This concentration change may be attributed to the aforementioned affinity affecting the assay. Therefore, the possibility of time‐dependent changes cannot be entirely ruled out.

In addition, it is important to note that once an infusion container has been punctured, the risk of bacterial contamination cannot be completely ruled out, even if the fluid was prepared in a clean bench environment. For these reasons, the data obtained in this study do not guarantee usability of the fluid up to 48 h after preparation. The actual expiration time should be determined based on the policies of each medical facility and the preparation environment.

This study has several important limitations. Our evaluation was conducted entirely in vitro, without assessment of clinical outcomes or patient safety. While we demonstrated compositional stability and electrolyte compatibility under laboratory conditions, the clinical efficacy and safety of this formulation during actual TPE procedures remain to be established. Future clinical studies are needed to determine whether the observed benefits translate into improved patient outcomes and reduced complications during TPE procedures.

## 5 Conclusion

Using a simple method to mix 25% HSA with RL, 10% sodium chloride, and 8.5% calcium gluconate hydrate solutions, we devised a TPE replacement fluid that was stable and demonstrated improved electrolyte compatibility with physiological levels.

## Author Contributions

Y.N. and Y.T. were involved conception and design of the study, literature research and writing of the manuscript. Y.N., Y.T., and T.M. performed the measurements. K.O. assisted with the measurements. K.O., H.N., D.K., and M.A. assisted with the conception and design of the study and writing of the manuscript. H.N., D.K., H. S., and M.A. were involved management of this study. The author(s) read and approved the final manuscript.

## Funding

The authors have nothing to report.

## Ethics Statement

The authors have nothing to report. This in vitro study did not involve human participants or patient samples.

## Consent

The authors have nothing to report. This in vitro study did not involve human subjects.

## Conflicts of Interest

This research was conducted as a joint project between Kumamoto University, Sojo University, and KM Biologics Co. Ltd., with research funding provided by KM Biologics Co. Ltd. Y.T., T.M., and H.N. are employees of KM Biologics Co. Ltd. The HSA solution used in this study was manufactured by KM Biologics Co. Ltd. The other authors declare no conflicts of interest.

## Data Availability

The datasets used and/or analyzed during the current study are available from the corresponding author on reasonable request.
